# Effects of genetic deletion versus pharmacological blockade of the LPA_1_ receptor on depression-like behaviour and related brain functional activity

**DOI:** 10.1242/dmm.035519

**Published:** 2018-09-10

**Authors:** Román Darío Moreno-Fernández, Andrea Nieto-Quero, Francisco Javier Gómez-Salas, Jerold Chun, Guillermo Estivill-Torrús, Fernando Rodríguez de Fonseca, Luis Javier Santín, Margarita Pérez-Martín, Carmen Pedraza

**Affiliations:** 1 Departamento de Psicobiologia y Metodologia en las CC, Instituto de Investigación Biomédica de Málaga (IBIMA), Universidad de Málaga, Málaga 29071, Spain; 2 Sanford Burnham Prebys Medical Discovery Institute, La Jolla, CA 92037, USA; 3 Unidad de Gestión Clínica de Neurociencias, Instituto de Investigación Biomédica de Málaga (IBIMA), Hospital Regional Universitario de Málaga, Málaga 29010, Spain; 4 Unidad de Gestión Clínica de Salud Mental, Instituto de Investigación Biomédica de Málaga (IBIMA), Hospital Regional Universitario de Málaga, Málaga 29010, Spain; 5 Departamento de Biología Celular, Genética y Fisiología. Facultad de Ciencias. Instituto de Investigación Biomédica de Málaga (IBIMA), Universidad de Málaga, Málaga 29071, Spain

**Keywords:** Animal models, LPA_1_ receptor, Genetic deletion, Antagonist, Functional brain mapping, Mixed anxiety-depression phenotype

## Abstract

Animal models of psychopathology are particularly useful for studying the neurobiology of depression and characterising the subtypes. Recently, our group was the first to identify a possible relationship between the LPA_1_ receptor and a mixed anxiety-depression phenotype. Specifically, maLPA_1_-null mice exhibited a phenotype characterised by depressive and anxious features. However, the constitutive lack of the gene encoding the LPA_1_ receptor (*Lpar1*) can induce compensatory mechanisms that might have resulted in the observed deficits. Therefore, in the present study, we have compared the impact of permanent loss and acute pharmacological inhibition of the LPA_1_ receptor on despair-like behaviours and on the functional brain map associated with these behaviours, as well as on the degree of functional connectivity among structures. Although the antagonist (intracerebroventricularly administered Ki16425) mimicked some, but not all, effects of genetic deletion of the LPA_1_ receptor on the results of behavioural tests and engaged different brain circuits, both treatments induced depression-like behaviours with an agitation component that was linked to functional changes in key brain regions involved in the stress response and emotional regulation. In addition, both Ki16425 treatment and LPA_1_ receptor deletion modified the functional brain maps in a way similar to the changes observed in depressed patients. In summary, the pharmacological and genetic approaches could ultimately assist in dissecting the function of the LPA_1_ receptor in emotional regulation and brain responses, and a combination of those approaches might provide researchers with an opportunity to develop useful drugs that target the LPA_1_ receptor as treatments for depression, mainly the anxious subtype.

This article has an associated First Person interview with the first author of the paper.

## Abstract (precis)

**Summary:** Animal models of psychopathology are useful for studying the neurobiology of depression. Here, we have assessed by pharmacological approach and knockout models the contribution of the LPA-LPA_1_ signalling pathway to anxious depression.

## INTRODUCTION

Animal models of psychopathology are widely used in neuroscience research (Nestler and Hyman, 2010). These models have not only enhanced our knowledge of the pathophysiological mechanisms of several mental disorders (Schmidt, 2011), but also provided opportunities to develop novel therapeutics (Czéh et al., 2016; Ma et al., 2017). In rodents, the depletion of key genes involved in the neurobiology of specific psychiatric diseases, i.e. the generation of knockout models, has become a common technique for elucidating the roles of specific genes and proteins in both the brain and behaviour (Sukoff Rizzo and Crawley, 2017). Such models hold tremendous promise for elucidating new pathways involved in disease, particularly when current treatment options are not completely effective.

Within the broad range of neuropsychiatric illnesses, depression is an illness for which studies aiming to clarify specific mechanisms are particularly needed, as these investigations will be crucial for developing effective therapeutic strategies and reducing the currently high rates of nonresponding patients (Willner and Belzung, 2015; Akil et al., 2018). In fact, >50% of depressed patients are not adequately treated by available therapies (Akil et al., 2018), and a high percentage of patients relapse after an initial response (Berwian et al., 2017) or develop treatment resistance over time (Akil et al., 2018). These low success rates are probably due to nonoptimal treatments. Although depression is diagnosed as a single entity, patients display a wide variety of symptoms, reflecting the great heterogeneity of this disorder. However, despite the heterogeneity of clinical patterns, few insights into the biological distinctions between subtypes of depression have been obtained (Krishnan and Nestler, 2011; Milaneschi et al., 2016; Drysdale et al., 2017; Cooper et al., 2018).

The identification of the dysfunctional brain circuits underlying specific subtypes of depression, as well as the changes in gene expression associated with these diseases, might lead to the generation of novel antidepressants tailored to specific patient populations whose depression involves distinct types of molecular and circuit dysfunctions (Akil et al., 2018; Cooper et al., 2018). A very common subtype of depression with a devastating impact is the comorbidity of anxiety and depression, known as ‘anxious depression’ (Fava et al., 2004; Braam et al., 2014; Bress et al., 2015). However, the underlying neurobiological mechanisms of this subtype of depression remain poorly understood. Studies aiming to reveal the unknown neurobiological bases of the mixed anxiety-depression phenotype are crucial for improving the diagnosis, prognosis and treatment (Ionescu et al., 2013).

Recently, our group was the first to identify a possible relationship between the LPA_1 _receptor [one of the six characterised G protein-coupled receptors (LPA_1-6_) through which lysophosphatidic acid (LPA) acts as an intracellular signalling molecule] and a mixed anxiety-depression phenotype, disclosing a possible neurobiological basis of this subtype of depression. Specifically, maLPA_1_-null mice exhibit a phenotype characterised by depressive (Moreno-Fernandez et al., 2017) and anxious features (Santín et al., 2009; Castilla-Ortega et al., 2010, 2013; Pedraza et al., 2014; Moreno-Fernández et al., 2017). Indeed, based on validity criteria (face, construct and predictive) and accumulating data from studies of animals lacking the LPA_1_ receptor, our group has proposed that maLPA_1_-null mice represent an animal model of anxious depression (Moreno-Fernández et al., 2017). In this sense, mice with this genotype exhibit abnormal emotional responses (Pedraza et al., 2014), cognitive alterations in hippocampus-dependent tasks (Castilla Ortega et al., 2011, 2014), anhedonia (Moreno-Fernández et al., 2017), anxiety (Santín et al., 2009), agitation, increased stress reactivity (Pedraza et al., 2014; Moreno-Fernández et al., 2017) dysfunctional coping in response to chronic stress (Castilla-Ortega et al., 2011) and fatigability, behaviours that are strongly associated with the psychopathological endophenotype of depression with anxious features (Moreno-Fernández et al., 2017). Furthermore, from a neurobiological perspective, adult maLPA_1_-null mice exhibit impairments in hippocampal neurogenesis (Matas-Rico et al., 2008), increased endocrine responses to emotional stimuli (Pedraza et al., 2014), hypoactivity and dysregulation of the normal function of the hypothalamic–pituitary–adrenal (HPA) axis after chronic stress (Castilla-Ortega et al., 2011), and dysfunctional changes among the highly interconnected ‘limbic’ regions and functional reorganisation of the brain network (Moreno-Fernández et al., 2017), all of which are factors that have also been strongly correlated with depression (Boucher et al., 2011) and anxiety (Hakamata et al., 2017). Moreover, both the behavioural symptoms and the abnormal patterns of brain connectivity in maLPA_1_-null mice are normalised by antidepressant treatment (Moreno-Fernández et al., 2017).

However, although knockout models are unquestionably indispensable instruments for establishing the neurobiological bases of depression, and maLPA_1_-null mice represent a good animal model of anxious depression, the constitutive lack of one target gene in general, or the LPA_1_ receptor gene (*Lpar1*) in particular, can induce compensatory mechanisms throughout the lifespan of the organism, causing several developmental neuroadaptations that might result in the observed deficits (El-Brolosy and Stainier, 2017). Therefore, validation with pharmacological studies is necessary to assess the contribution of the LPA-LPA_1_ signalling pathway to a specific disease.

Notably, pharmacological manipulation of the LPA_1_ receptor has also been linked to various mood-related effects (Castilla-Ortega et al., 2014; Kim et al., 2017). Interestingly, some of the effects of LPA_1_ receptor antagonism by Ki16425 mimic the behavioural features of maLPA_1_-null mice in fear extinction tests (Pedraza et al., 2014), and brain and behavioural impairments associated with alcohol consumption (Castilla-Ortega et al., 2016). Here, we sought to explore the possible parallelism between the permanent loss of the LPA_1_ receptor and the pharmacological inhibition of the LPA_1_ receptor by its antagonist Ki16425 in the context of behavioural despair- and stress-induced brain activation. Therefore, we employed two behavioural despair tasks; namely, the forced swim test (FST) and the tail suspension test (TST), along with pharmacological and genetic approaches. Additionally, we studied c-Fos immunoreactivity in the corticolimbic and extralimbic circuits, which are involved in emotional regulation, to examine the map of functional interregional activation and the degree of connectivity to identify differences in brain responses to acute stress (FST).

## RESULTS

### Behavioural despair

#### FST

**Fig. 1.**
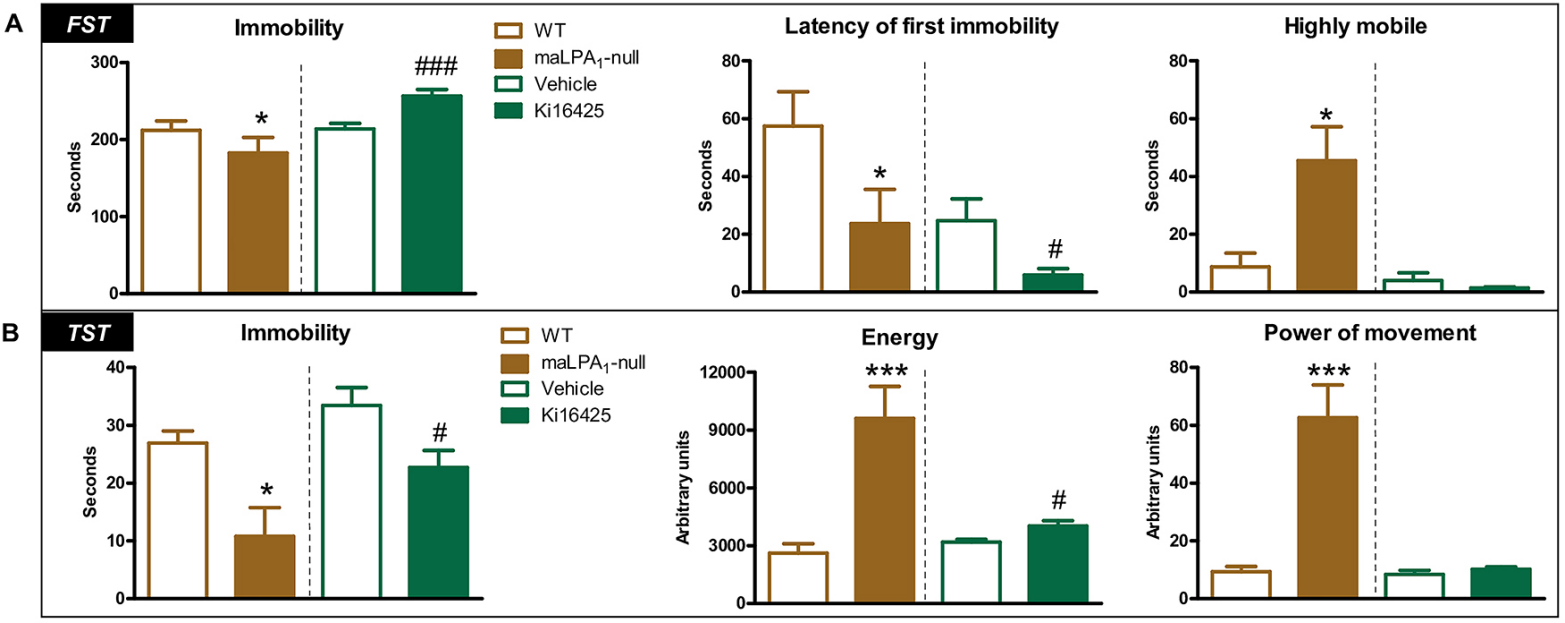
**The LPA_1_ receptor is required for normal stress-coping responses.** (A) The genetic deletion of the LPA_1_ receptor and functional antagonism exerted opposite effects on the immobility time in the FST. However, both groups showed reduced latencies to become immobile. An increase in the highly mobile time was only observed in maLPA_1_-null mice. WT, *n*=6; maLPA_1_-null, *n*=6; veh, *n*=11; Ki16425, *n*=17. (B) In the TST, both maLPA_1_-null mice and Ki16425-treated mice showed reduced immobility times and incremented energy compared with control groups. WT, *n*=7; maLPA_1_-null, *n*=7; veh, *n*=7; Ki16425 *n*=6. Student’s *t*-test was used for statistical analysis; *P<0.05, ***P<0.005 compared with WT; ^#^*P*<0.05, ^###^*P*<0.005 compared with vehicle group. FST, forced swim test; TST, tail suspension test.

Student's *t*-test (*t*) and Welch's *t*-test (*t_w_*) revealed significant differences between treatments. Specifically, the duration of immobility was significantly reduced in the maLPA_1_-null mice compared with that in the wild-type (WT) mice [*t*(9)=3.29, *P*<0.05; the number in the parentheses represents the degrees of freedom of the analysis] but was increased in Ki16425-treated compared with vehicle solution (veh)-treated mice [*t*(25)=–3.10, *P*<0.005]. Nevertheless, both maLPA_1_-null and antagonist-treated mice showed a reduced latency to the first immobility period [*t*(9)=2.54, *P*<0.05, WT versus maLPA_1_-null mice; and *t_w_*(25)=2.01, *P*<0.05, veh- versus Ki16425-treated animals]. The latency to immobility has been reported to be a more sensitive measure of depression-like behaviours than the duration of immobility (Castagné et al., 2009). An increase in climbing/struggling behaviour, measured as high mobility, was observed in maLPA_1_-null mice [*t*(9)=−2.90, *P*<0.05]. The pharmacological blockade of the LPA_1_ receptor did not induce any changes in these parameters (Fig. 1A).

#### TST

According to our data, both maLPA_1_-null mice and Ki16425-infused mice showed a reduced immobility time [*t_w_*(11)=2.01, *P*<0.05; WT versus maLPA_1_-null mice, and *t*(9)=2.38, *P*<0.05; veh- versus Ki16425-treated animals] and increased energy [*t_w_*(11)=−4.40, *P*<0.005, WT versus maLPA_1_-null mice; and *t*(9)=2.38, *P*<0.05, veh- versus Ki16425-treated animals] compared with their respective control groups. Additionally, maLPA_1_-null mice showed increased power of movement compared with WT mice [*t_w_*(11)=−4.75, *P*<0.005] (Fig. 1B).

### Determination of brain activation

After the FST, c-Fos-immunoreactive (IR) cells were counted in the dorsal and ventral (d/v) hippocampus (Hipp) [i.e. d/v dentate gyrus (DG), d/v cornu ammonis 1 (CA1) and d/v cornu ammonis 3 (CA3)], the medial prefrontal cortex (mPFC) [including both the prelimbic (PL) and infralimbic (IL) cortices], the central and basolateral amygdala (CeA and BLA, respectively), the nucleus accumbens (NAc; both the core and the shell), the habenula [lateral (LHb) and medial (mHb)], the ventral tegmental area (VTA), the dorsal raphe nucleus (DRN), the paraventricular nucleus of the hypothalamus (PVN), and the d/v periaqueductal grey (PAG).

**Fig. 2.**
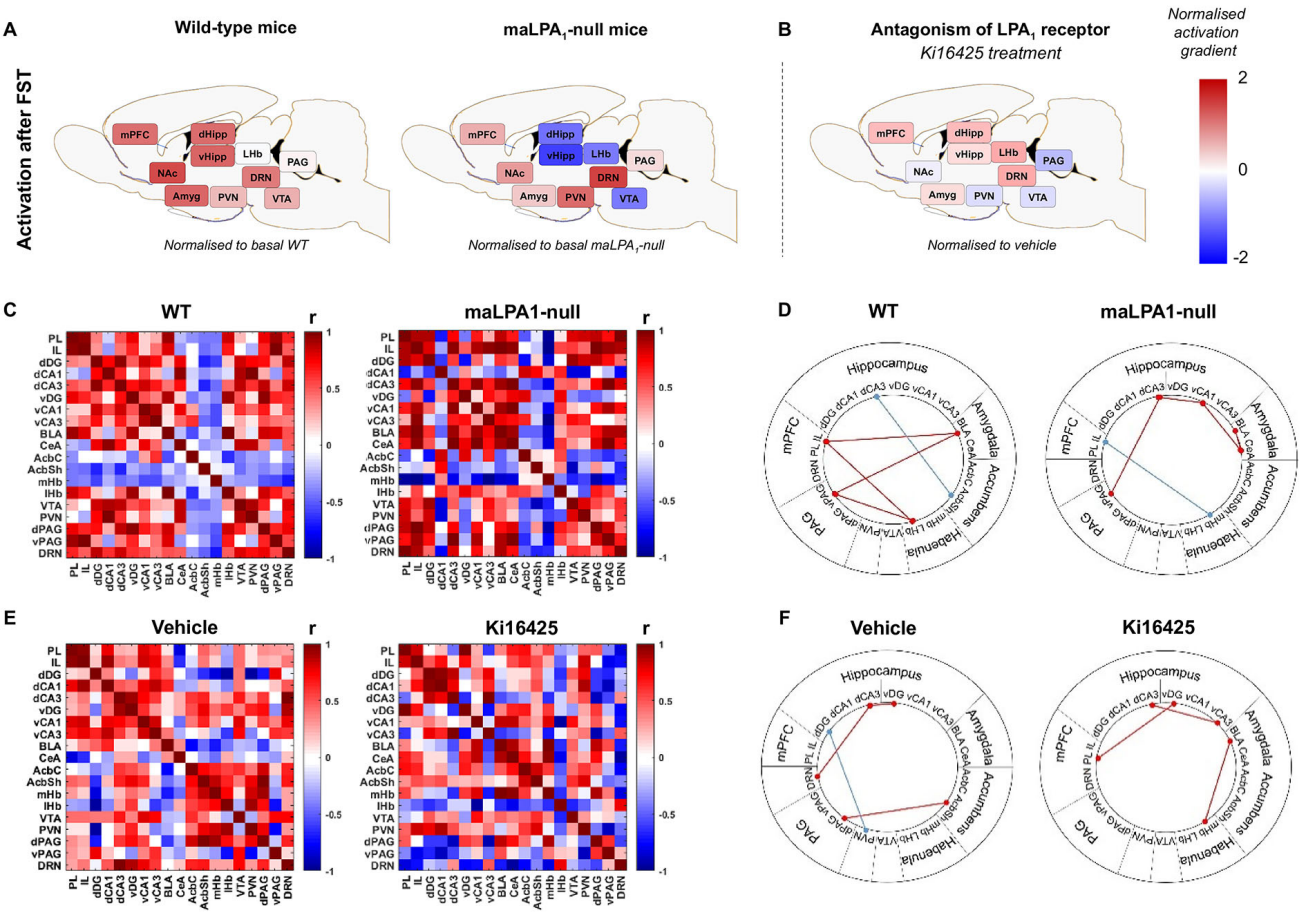
**Changes in brain activation and connectivity observed after the FST depend on the LPA_1_ receptor.** (A,B). Representation of the functional activation patterns in the experimental groups. Data from WT and maLPA_1_-null mice were normalised to the respective baseline control groups (A). Data from Ki16425-treated animals were normalised to those from the veh-treated group (B). (C-F) Additionally, interregional correlation maps for both genotypes (C) and i.c.v.-treated mice (E) are displayed, along with diagrams showing strong, significant correlations. (D,F; r≥±0.8; *P*≤0.005). WT, *n*=5; maLPA_1_-null, *n*=5; veh, *n*=5; Ki16425, *n*=5. AcbC, nucleus accumbens core; AcbSh, nucleus accumbens shell; BLA, basolateral amygdala; CeA, central amygdala; DRN, dorsal raphe nucleus; d/vCA1, dorsal/ventral cornu ammonis 1; d/vCA3, dorsal/ventral cornu ammonis 3; d/vDG, dorsal/ventral dentate gyrus; d/vPAG, dorsal/ventral periaqueductal grey matter; FST, forced swim test; IL, infralimbic cortex; LHb, lateral habenula; mHb, medial habenula; mPFC, medial prefrontal cortex; NAc, nucleus accumbens; PL, prelimbic cortex; PVN, paraventricular nucleus; VTA, ventral tegmental area.

The estimated numerical density of c-Fos^+^ cells (c-Fos per mm^3^) in the DRN [*t*(10)=−2.29, *P*<0.05], PVN [*t*(10)=−2.15, *P*<0.05] and dPAG [*t*(9)=−2.44, *P*<0.05] was greater in the maLPA_1_-null mice than in the WT mice; the opposite trend was observed for the dorsal hippocampus [*t*(10)=1.94, *P*=0.08 for dDG; *t*(10)=3.57, *P*<0.01 for dCA3; Fig. S2]. In contrast, the Ki16425-treated group exhibited increased activity in the dorsal hippocampus after completing the FST [*t*(8)=−2.12, *P*=0.06 in dCA1]. Moreover, enhanced activity in the LHb [*t*(8)=−2.62, *P*<0.05] and reduced activity in the nucleus accumbens core [*t*(8)=1.99, *P*=0.08] and dPAG [*t*(8)=3.24, *P*<0.01] were also observed in antagonist-treated animals under stress. The data indicated clear differences in brain functional activity induced by FST in animals in which the LPA_1_ receptor was inactivated by different procedures; these changes might underlie the differences in behaviour during the FST (Fig. 2A,B; Figs S1-S3).

Because depression is associated with functional brain abnormalities, including regional connectivity (Ressler and Mayberg, 2007), the neural network organisation in the four groups was explored. We analysed the complete matrices of correlations between brain regions in each group of mice after the FST (Fig. 2B,D). The computation of different patterns of co-activation after the behavioural test is important for identifying the differences in the brain networks engaged in coping with stress among the experimental groups. As revealed by the data, regional immediate early gene expression exhibited a LPA_1_-dependent, patterned relationship with stress-coping behaviours (Fig. 2B-E). The patterns of connectivity between brain regions differed as a function of genotype or pharmacological treatment after behavioural tests.

**Fig. 3.**
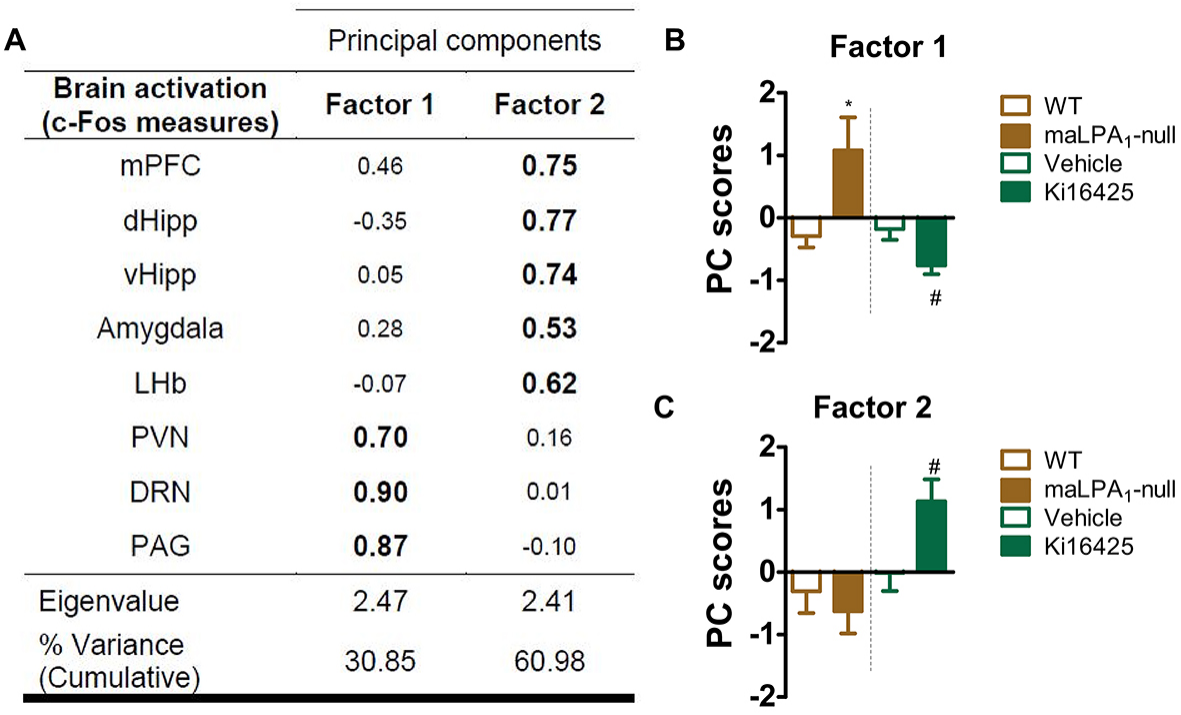
**PCA** **reveals different routes of brain activation between maLPA_1_-null and Ki16425-treated mice.** (A) Interpretable factor loadings are shown in bold. Variables with negative scores are inversely correlated to the factor. Rotation method: varimax with Kaiser normalisation. Rotation converged in three iterations. KMO=0.55; χ²=63.2; *P*<0.001. (B,C) Representative images of factor scores for factor 1 (B) and factor 2 (C). maLPA_1_-null mice showed the highest score for factor 1 (PVN, DRN and PAG); Ki16425-treated mice showed the highest for factor 2 mPFC (d/vHipp, amygdala and LHb). Student’s *t*-test was used for statistical analysis; *P<0.05 compared with WT; ^#^*P*<0.05 compared with veh-treated group. DRN, dorsal raphe nucleus; d/vHipp, dorsal/ventral hippocampus; FST, forced swim test; IL, infralimbic cortex; LHb, lateral habenula; mHb, medial habenula; mPFC, medial prefrontal cortex; PAG, periaqueductal grey matter; PVN, paraventricular nucleus.

A principal component analysis (PCA) was conducted to study the relationships among brain structures and established what appears to be a functional network engaged by behaviour. PCA with variance-maximising (varimax) rotation revealed a two-component solution accounting for 61% of the total variance. Strong correlations were observed among PVN, DRN and PAG functional activity (factor 1, which was named ‘extralimbic regions’) and among the mPFC, d/vHipp, amygdala and LHb (factor 2, which was named ‘limbic regions’) (Fig. 3A). Finally, factor scores were calculated and compared among groups. Based on Student's *t*-tests, in the FST, in comparison with their respective control group, maLPA_1_-null mice exhibited enhanced activation of the extralimbic network [*t*(10)=−2.46, *P*<0.05], whereas antagonist-treated animals showed reduced activation of the ‘extralimbic network’ and increased activity in the ‘limbic network’ [*t*(8)=2.59, *P*<0.05; *t*(10)=−2.50, *P*<0.05, respectively] (Fig. 3B,C).

**Fig. 4.**
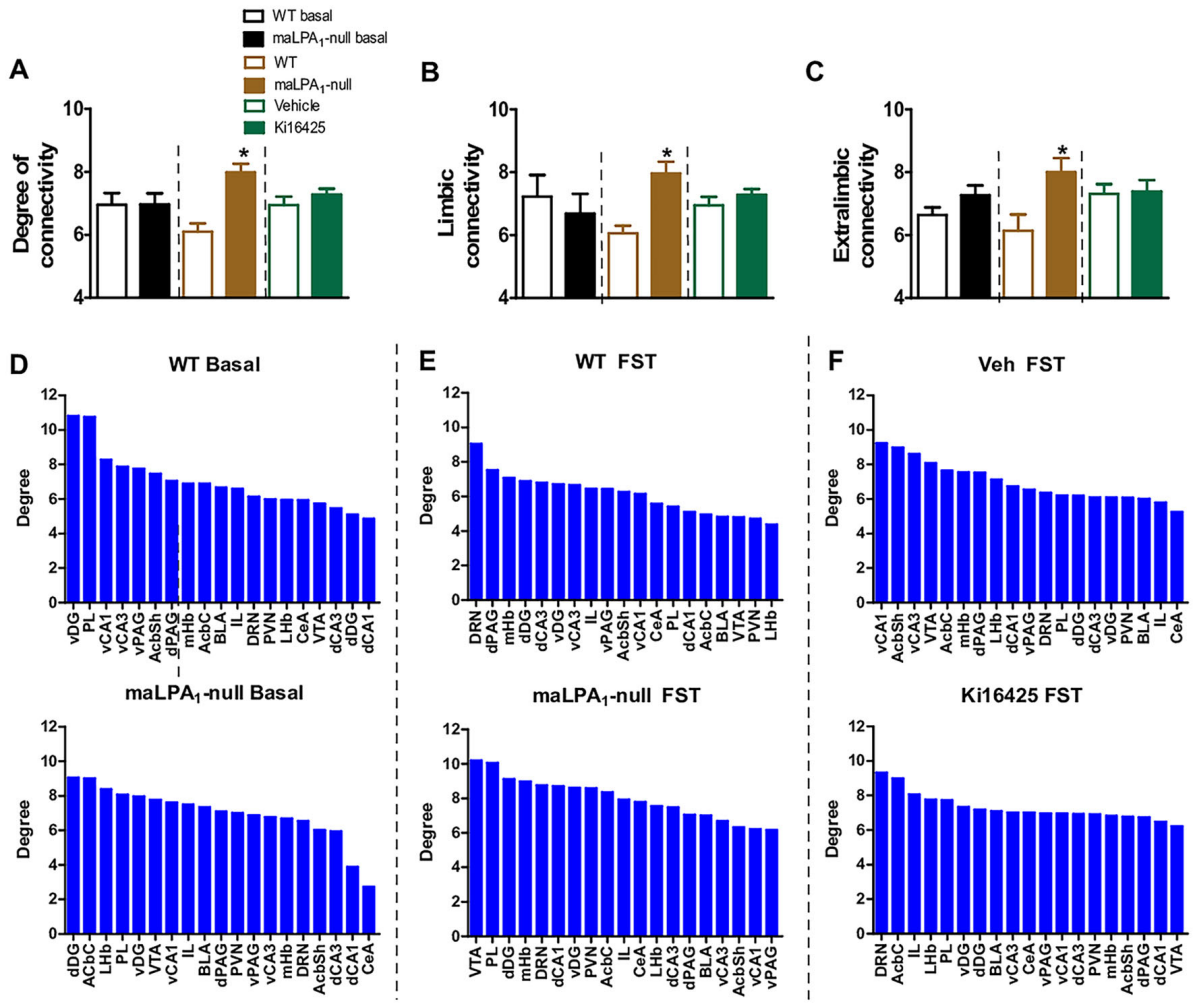
**Treatments modified the degree of connectivity between the regions studied.** (A) Global degree of connectivity among all examined regions. At baseline, differences were not observed between genotypes. After the FST, maLPA_1_-null mice displayed the highest degree of connectivity. (B) Degree of connectivity of limbic regions (PL, IL, BLA, CeA, dDG, dCA1, dCA3, vDG, vCA1, vCA3). (C) Degree of connectivity of extralimbic regions (mHb, LHb, VTA, PVN, dPAG, vPAG, DRN). (D-F) Brain regions ranked in descending order by the degree of connectivity in each group. Baseline WT=5; baseline maLPA1-null mice=6; WT, *n*=5; maLPA_1_-null, *n*=5; veh, *n*=5; Ki16425, *n*=5. Student’s *t*-test was used for statistical analysis; *P<0.05 compared with WT. AcbC, nucleus accumbens core; AcbSh, nucleus accumbens shell; BLA, basolateral amygdala; CeA, central amygdala; DRN, dorsal raphe nucleus; d/vCA1, cornu ammonis 1; d/vCA3 dorsal/ventral cornu ammonis 3; d/vDG, dorsal/ventral dentate gyrus; d/vPAG dorsal/ventral periaqueductal grey matter; IL, infralimbic cortex; LHb, lateral habenula; mHb, medial habenula; PL, prelimbic cortex; PVN, paraventricular nucleus of the hypothalamus; VTA, ventral tegmental area.

Furthermore, the degree of connectivity was calculated for all structures analysed and independently for limbic and extralimbic regions according to the factors obtained in the PCA (Fig. 4A-C). No differences in the baseline degree of connectivity were observed between genotypes. However, after the FST, the genetic deletion of the LPA_1_ receptor increased connectivity levels globally [*t*(36)=−4,86, *P*<0.001], and in the limbic regions and extralimbic network [*t*(18)=−4.28, *P*<0.005; *t*(16)=−2.73, *P*<0.05, respectively], compared with WT animals. Additionally, the identification of highly connected regions revealed differences between treatments (Fig. 4D).

Notably, the identification of sets of activated regions considered ‘networks’ does not necessarily mean that the regions are directly connected from a neuroanatomical perspective.

## DISCUSSION

Animal models are essential to elucidate the neurobiology of depression, to test novel neurobiological hypotheses regarding depression-related outcomes, and to enable the identification of new targets for improved and targeted therapy. We have previously proposed the possible participation of the LPA_1_ receptor in affective disorder; if dysfunctional, this receptor might induce vulnerability to the development of anxious depression (Moreno-Fernández et al., 2017), a prominent subtype of depression with unknown neurobiological bases and poor treatment responses compared with anxiety or depression alone (Richards et al., 2016). Because the possible participation of the LPA_1_ receptor in depression-like disorders has been proposed in a knockout model with a constitutive lifelong lack of this receptor, validation with pharmacological studies is necessary to assess the contribution of the LPA-LPA_1_ signalling pathway to mood disorders. Therefore, in this study, we compared the behavioural outcomes and brain functional activity of mice with a genetic deletion and acute pharmacological blockade of the LPA_1_ receptor using two stress tests that are widely employed to monitor helplessness and study depression in animal models, i.e. the FST and TST.

Regarding the comparison of behavioural responses exhibited by animals in the FST and TST, although some responses in both tests were similar between treatments [latency to first immobility in the FST and reduced immobility and increased energy in the TST (Fig. 1A,B)], other responses were markedly different. In particular, maLPA_1_-null mice exhibited a reduction in total immobility time in the FST, whereas this variable was increased in animals treated with an antagonist. Many behavioural reactions in behavioural despair tests, such as the TST and the FST, are considered to reflect pathological coping mechanisms in response to an aversive environment (Boulle et al., 2014). In the FST, either the increased immobility time or reduced immobility with the addition of increased struggling/climbing behaviour are interpreted as reflecting depression-like behaviours in rodents, particularly when either reaction is coupled with a reduced latency to the first immobility [inferred as a reduction in intrinsic motivation to escape the situation (Castagné et al., 2009)]. Therefore, both procedures are postulated to induce depression-like behaviours. However, maLPA_1_-null mice exhibited an excessive emotional response, showing anxiogenic-like reactions in both tests (Anyan and Amir, 2018), as previously described (Moreno-Fernández et al., 2017), reflecting a comorbid phenotype between depression and anxiety-like behaviours (Anyan and Amir, 2018; Moreno-Fernández et al., 2017). Antagonist-treated animals only exhibited anxious behaviours in the TST. The divergent behaviours of the antagonist-treated group in the FST and TST could be attributed to procedural differences in both tests, particularly because the TST is not a dry-land version of the FST (Cryan et al., 2005) and the neurobiological pathways involved in these two models are likely not identical (Bai et al., 2001; Yoshikawa et al., 2002). Notably, several compounds, such as rolipram, a selective phosphodiesterase-4 inhibitor, and levoprotiline, which acts as an antihistamine, reduce immobility in the FST but are reportedly inactive in the TST (Porsolt and Lenegre, 1992). In contrast, selective serotonin reuptake inhibitors reduce immobility more consistently in the TST than in the FST (reviewed in Porsolt and Lenegre, 1992). Moreover, a quantitative trait loci analysis using C57BL/6 mice has identified different genes that might contribute to different immobility responses in the TST and FST (Yoshikawa et al., 2002). Overall, both methods of inactivating the LPA_1_ receptor induced depression-like behaviours but engaged partially different dysfunctional mechanisms to cope with the aversive situation.

On the other hand, although the neurobiological substrate of anxious depression has not yet been completely determined, the hippocampus is, from a neuroanatomical perspective, one of the most important regions for understanding both categorical and dimensional aspects of anxiety and depression, and is one of the most important hubs in the functional connectome involved in emotional regulation (Roberts et al., 2018). This region, which is dysfunctional in maLPA_1_-null mice (Matas-Rico et al., 2008; Castilla-Ortega et al., 2011; Musazzi et al., 2011; Peñalver et al., 2017), is consistently implicated in anxiety- and depression-relevant processes, such as threat processing, reward and motivation, and affects regulation and memory (Oathes et al., 2015). In our study, the hippocampi of the maLPA_1_-null mice reacted with lower activity after the FST than under basal conditions, and with lower activity than WT animals after the FST, whereas Ki16425-treated animals showed an increased level of functional activity compared with the veh-treated group. These opposite responses of the hippocampus could partially explain the observed differences in FST responses. In maLPA_1_-null mice, reduced hippocampal activation has been coupled with hyperactivity of the PVN, which is associated with stress-induced neuroendocrine and molecular responses of the HPA axis, and can affect the ability to regulate emotion (Pedraza et al., 2014; Moreno-Fernández et al., 2017), and induce a negative mood bias that results in long-term depressive and anxious symptoms (Cai et al., 2015; Ebner and Singewald, 2017). Additionally, after the FST, maLPA_1_-null mice displayed enhanced activation of the DRN, a region essential for mediating aversion (Harrison et al., 2003; Elizalde et al., 2010; Disner et al., 2011; Moreno-Fernández et al., 2017). In the antagonist-treated group, the activation of the hippocampus was accompanied by augmented activity of the LHb, which has been correlated with aversive states (Margolis and Fields, 2016), and might be at least part of the substrate responsible for the increased immobility time (Yang et al., 2008). In addition, after the FST, a reduction in PAG activity was observed that could be linked to negative affect (Miczek et al., 1999; Mickley et al., 2011; Coulombe et al., 2017). Conversely, in WT animals, stress engaged all the limbic and almost all extralimbic regions examined, except for the LHb. The PAG was minimally engaged by the FST. Therefore, the changes in functional activity induced by behaviour in animals with genetic deletion or pharmacological blockade of LPA_1_ receptors could be related to dysfunctional emotional outcomes, although different changes in functional brain activity are involved.

However, the brain is organised into a complex network of interconnections that enables emotional information processing and behaviour regulation, among other functions. Functional connectivity can be defined by the functional co-activation of spatially distributed brain regions (Tadayonnejad and Ajilore, 2014). Along with functional changes in the brain, depression and anxiety are associated with disrupted brain networks (Gong and He, 2015; Tovote et al., 2015; Qiao et al., 2017), which are responsible for the maladaptive processes underlying these diseases (Hakamata et al., 2017; Rosenbaum et al., 2017). Both treatments induced changes in the functional brain map compared with their respective control groups (WT or veh), although the treatments engaged different brain regions, as confirmed by the factors associated with brain activity in the PCA analyses. Thus, in null mice, increased activity of the ‘extralimbic network’ (composed of the PVN, DRN and PAG) was observed after the FST. This network, which has been linked to anxiety and stress responses (Waselus et al., 2011), might explain the augmented stress reactivity observed in this genotype during behavioural tasks. Conversely, in antagonist-treated animals, the FST engaged the ‘limbic network’, but nevertheless reduced the activity of the ‘extralimbic network’. This finding might explain why an increase in the immobility time, but no change in climbing/struggling time, were observed in antagonist-treated animals. Although the FST exerted different effects on the brain network in response to each treatment, changes in the functional map observed in maLPA_1_-null mice and antagonist-treated group have been linked to mood dysregulation. Therefore, elevated emotional reactivity to stressors could indicate a diminished ability to cope with challenges and might increase vulnerability to a depressed mood and anxiety (Sandi and Richter-Levin, 2009). Concurrently, excessive limbic activation might affect the ability to regulate emotion (Pedraza et al., 2014) and cause a negative mood bias and maladaptive processing of emotional stimuli, resulting in long-term depressive dysfunction and symptoms of anxiety (Disner et al., 2011).

Moreover, because abnormal communication among brain regions has been observed in both patients with depression (Sheline et al., 2010; Kaiser et al., 2015; Rosenbaum et al., 2017) and those with anxiety disorders (Pannekoek et al., 2013a,b), and correlates with changes in emotional state (Cribben et al., 2012), we calculated the global connectivity among the examined structures, as well as between limbic and extralimbic regions involved in depression and anxiety. Under basal conditions, no differences in the degree of connectivity were observed between WT and maLPA_1_-null mice. However, animals lacking the LPA_1_ receptor showed increased degrees of global, limbic and extralimbic connectivity after the FST compared with WT animals, whereas the FST had no effect on the degree of connectivity in antagonist-treated animals. As depression-related and anxiety-related (Pannekoek et al., 2013a,b) increases in connectivity have been identified between networks responsible for emotional regulation (Sheline et al., 2010), the enhanced functional connectivity observed in maLPA_1_-null mice might underlie some aspects of emotional dysregulation and the depressive and anxious phenotype observed here. On the other hand, the identification of highly interconnected regions revealed that both treatments modified the rank of highly interconnected regions. Highly interconnected regions can disproportionately influence the function of a network (Wheeler et al., 2013), particularly when the most highly interconnected areas are abnormally engaged by behaviour, as is the case for the FST in response to both treatments. For example, in maLPA_1_-null mice, the functionally impaired hippocampus might exert a substantial impact on the limbic network; in contrast, in the antagonist-treated group, the LHb, a region essential for mediating aversion (Stamatakis and Stuber, 2012), might influence limbic network function. Nevertheless, although changes in these highly interconnected regions could be involved in abnormal test outcomes after both forms of LPA_1_ receptor inactivation, future research should further establish the relationship between changes in the ranking of highly interconnected regions and behavioural outcomes. Undoubtedly, these treatments produced impaired reorganisation of the brain network, based on the data obtained from investigations of functional brain activation and its correlation with behavioural outcomes.

In summary, these data provide evidence that the LPA_1_ receptor is involved in adaptive coping strategies, revealing that LPA_1_ pathways might be critical components of the pathogenesis of depression. However, the antagonist mimics some, but not all, effects of genetic deletion of the LPA_1_ receptor. The observed mismatch between the phenotype of the mouse lacking the LPA_1_ receptor, and the effects of the antagonist on normal animals, were explained as follows: (1) the phenotype of the maLPA_1_-null mouse was due to adaptive changes rather than a lack of receptors in the adult animals, such as changes in other neurotransmitter systems, i.e. glutamatergic systems (Blanco et al., 2012); (2) chronic dosing rather than acute dosing is required; (3) whole-body LPA_1_ deletion exerted greater effects than brain-restricted inhibition of LPA_1_ by intracerebroventricular (i.c.v.) injections of Ki16425; or (4) a lack of selectivity of Ki16425 for LPA_1_. According to the study by Ohta et al. in, 2003, the K_i_ of Ki16425 is three- and 20-fold higher for LPA_3_ and LPA_2_, respectively, than for LPA_1_. Therefore, the effects of Ki16425 might at least partially result from LPA_2_ or LPA_3_ inhibition. Nevertheless, the low expression of LPA_2_ and LPA_3_ receptors in the mouse brain limits this possibility (Choi et al., 2010). Both procedures undoubtedly induced depression-like behaviours with an agitation component (in both examined tests in maLPA_1_-null mice and in the TST for the antagonist-treated group), and although these treatments engaged different brain circuits, both pathologically altered the functional brain maps involved in emotional regulation and stress responses, the deregulation of which may be implicated in the development of depressive and anxious symptoms.

In conclusion, the pharmacological and genetic approaches reported here might play complementary roles in dissecting the function of the LPA_1_ receptor in modulating behavioural and brain responses. Pharmacology can reveal the function of this receptor when the relevant circuitry is acutely blocked, while the absence of a gene in the maLPA_1_-null model might illustrate the impact of the receptor on neural plasticity, homeostasis and animal models of genetic diseases. The present findings extend the former conclusions that maLPA_1_-null mice represent a good animal model of anxious depression, corroborating previous experimental evidence. Moreover, our data provide evidence supporting the hypothesis that the LPA_1_ receptor plays an essential role in regulating emotions and mood. Converging evidence generated from the two approaches strengthens the case for developing LPA_1_-receptor-targeted drugs that could be useful treatments for depression, mainly the anxious depression subtype.

## MATERIALS AND METHODS

### Animals

All experiments were performed using mice (*Mus musculus*) on a mixed C57BL/6×129X1/SvJ background. For studies involving maLPA_1_-null mice, trials were conducted on age-matched male WT and maLPA_1_-null homozygous littermates that were ∼3 months old. The maLPA_1_-null mouse (termed the maLPA_1_-Málaga variant of the LPA_1_-null mouse), was spontaneously derived during the expansion of the original colony (Contos et al., 2000) by crossing heterozygous founder parents (maintained on the original hybrid C57BL/6J6×1291/SvJ background) (Estivill-Torrús et al., 2008).

Pharmacological treatments (veh and Ki16425) were administered to 3-month-old B6129SF2/J male mice obtained from The Jackson Laboratory (Bar Harbor, ME, USA). B6129SF2/J mice are hybrid mice on a mixed C57BL/6J×129S1/SvImJ background and are generally considered acceptable controls for mutant mice on mixed B6/129 backgrounds. The experiments were performed in two independent batches of WT/null (*n*≥6 animals per genotype and behavioural test) and veh-/antagonist-treated mice (*n*≥6 for TST and n≥11 for FST), owing to the usual difficulty in obtaining an exact paired number of null mice at similar time points (limited breeding of the maLPA1-null colony). The experimental results were similar between batches.

Mice were housed in groups of four animals per cage with water and food provided *ad libitum*. Experiments were conducted between 09:00 h and 15:00 h [12-h light/dark cycle (lights on at 08:00 h)]. B6129SF2/J mice were housed and bred in the same room as animals with the genetic deletion of the LPA_1_ receptor, in contiguous cages to enable visual, auditory and olfactory contact. Only tactile contact was limited.

All procedures were performed in compliance with European animal research laws [European Communities Council Directives 2010/63/UE, 90/219/CEE, Regulation (EC) No. 1946/2003] and the Spanish National Guidelines for Animal Experimentation (Real Decreto 53/2013), approved by the Ethics Committee of Malaga University (CEUMA: 1-2015-A), and supported by the Andalusian Ministry of Agriculture and Fishing (08-7-15-273).

### Pharmacological treatment

We studied the effect of acute administration of a selective LPA_1_ receptor antagonist on behavioural despair by i.c.v. injecting Ki16425 [(ApexBio, Houston, TX, USA) (400 nM)] dissolved in a vehicle solution (veh) [3% fatty acid-free bovine serum albumin (Sigma-Aldrich, St Louis, MO, USA)/PBS] 30 min before the FST or the TST. This dose of Ki16425 was selected based on its K_i_ value of 0.34 μM for the LPA_1_ receptor (Ohta et al., 2003), and injections were administered in the right lateral cerebral ventricle (2 μl/mouse), according to the method reported by Ocaña et al. (1995) using a 10 μl syringe with a sleeve attached to the needle, which allows perforation of the skull to a depth of less than 3 mm, as detailed in Pedraza et al. (2014).

### Behavioural despair tests

#### FST

The protocol used in the present study was the ‘modified version of the FST’ (Cryan et al., 2002), adapted from the original version described by Porsolt et al. (1977, 1978). Swimming sessions were conducted by placing animals in individual Plexiglas cylinders (Malaga, Spain; 26×10 cm), containing 15 cm deep water at 23-24°C. The 6 min test session was recorded using a video camera positioned in front of the tank. The test was conducted as described in Moreno-Fernández et al. (2017). As previously described (Juszczak et al., 2008; Wong et al., 2017), automatic measurements of floating/immobility (immobility threshold of <12%) and climbing/struggling (threshold of >18.5%) were obtained twice per second using the software EthoVision XT 12 (Noldus Information Technology, Wageningen, The Netherlands).

#### TST

An automatic TST (Panlab Harvard Apparatus, Panlab, Barcelona, Spain) was employed using previously described methods (Moreno-Fernández et al., 2017). Mice were suspended by their tails fixed with adhesive tape to a hook that was coupled to a computer-assisted device for measuring movement for 6 min (Panlab Harvard Apparatus). Immobility (defined as when the animal hung passively without limb movement), energy and power of movement (PM), which are considered additional parameters of the intensity of climbing/struggling, were assessed. All parameters were analysed using a computerised system connected to the apparatus.

### Histology

#### Immunohistochemistry

Ninety minutes after completing the behavioural test (FST), mice were intracardially perfused with 4% (w/v) paraformaldehyde in 0.1 M phosphate buffer under anaesthesia. Brains were postfixed, immersed in a 30% sucrose solution to cryoprotect the tissues, and cut into coronal sections (30 μm) using a freezing microtome.

Immunohistochemistry was performed on free-floating brain sections [*n*=5 per experimental group (WT/maLPA_1_ and veh/Ki16425)]. A mouse anti-c-Fos antibody (1:1000; sc-52, Lot B0111, Santa Cruz Biotechnology, Santa Cruz, CA, USA) was used to detect neuronal activity. The secondary antibody was a biotin-conjugated swine anti-rabbit IgG (1:500; E0353, Lot 00088339, Dako, Glostrup, Denmark). Subsequently, sections were incubated with peroxidase-conjugated ExtrAvidin (1:1000, Sigma-Aldrich) with diaminobenzidine (in 0.01% hydrogen peroxide in 0.1 M phosphate buffer) as the chromogen. Negative controls were conducted for each antibody by omitting the primary antibody.

#### Cell counting

Cell counts in the interconnected limbic and extralimbic regions that have been implicated in depression and anxiety were recorded.

c-Fos-IR cells were counted in the d/vHipp (i.e. d/vDG, d/vCA1 and d/vCA3), mPFC (PL and IL cortices), CeA and BLA, NAc (both the core and the shell), LHb and mHb, VTA, DRN, PVN and d/vPAG. For this purpose, each region of interest was delineated according to the criteria by Paxinos and Franklin (2001). The coordinates along the anteroposterior axis were as follows: d/vHipp, from −1.22 to −2.18 and −2.82 to −3.40, respectively; mPFC, from +1.98 to +1.54; CeA and BLA, from −1.06 to −1.70; NAc, from +1.70 to +1.20; LHb and mHb, from −0.94 to −2.18; VTA, from −2.92 to −3.88; DRN, from −4.36 to −4.90; PVN, from −0.70 to −0.94; and d/vPAG, from −4.16 to −4.90. Additionally, the baseline count of c-Fos immunoreactivity in naïve WT and maLPA1-null mice was used.

An Olympus BX51 microscope (Olympus, Glostrup, Denmark) was interfaced with a computer and a colour JVC digital video camera. The number of c-Fos-IR nuclei was counted using the CAST-Grid software package (Olympus). The data were quantified by performing systematic sampling in each region using the following formula:

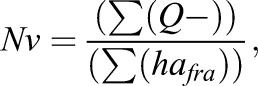
where *Nv* is the mean number of c-Fos-IR nuclei per volume unit (mm^3^), *Q−* is the total number of c-Fos-IR nuclei counted in all frames examined, *a_fra_* is the area of the sampling frames used and *h* is the thickness of the sections. At least three sections from each region were counted per animal. Because of the small volume of the DRN, PVN, habenula and PAG nuclei, the whole volume of each slice (100%) was counted. Otherwise, 50% of the total volume of each region was analysed using the CAST-Grid system. For this purpose, the number and distribution of the counting frames were randomly placed over half of the selected brain region. The resulting densities were averaged to obtain the mean number of c-Fos-IR cells (Nv) per volume unit. In addition, due to baseline differences in c-Fos counts between naïve WT and maLPA_1_-null mice (Moreno-Fernández et al., 2017), the data obtained from the WT and maLPA_1_-null group after the FST were normalised to the data obtained from the WT and maLPA_1_-null mice at baseline, respectively [(individual c-Fos count−control mean c-Fos count)/overall s.d.] (Ritov et al., 2015), as previously described (Moreno-Fernández et al., 2017). Additionally, the data obtained from the Ki16425 group after the FST were normalised to the veh control groups, as described.

### Statistical analysis

All results are presented as the mean±s.e.m.; *P*≤0.05 was considered statistically significant. Extreme upper or lower values were identified as outliers in box plots and discarded from the statistical analysis.

#### Behavioural tests

The duration of behaviours exhibited by animals of each group during the FST and TST and the latency of the first immobility period, defined as the time from start to the first bout of immobility lasting longer than 2 s, were analysed using Student's *t*-test (*t*). Levene's test was used to test the assumption of homogeneity of variance. Welch's *t*-test (*t_w_*) was performed when two samples have unequal variances.

#### Brain functional activity and connectivity analysis

Student's *t*-test was used to examine behaviour-induced functional differences between WT and maLPA_1_-null mice and between veh- and Ki16425-treated mice.

We conducted correlation analyses between behaviours and regional c-Fos expression to assess potential differences in functional brain activity related to the FST performance between groups. For functional brain mapping of each group of animals (WT, maLPA_1_-null, veh and Ki16425), all possible pairwise correlations between the c-Fos signal intensities in the 19 examined regions involved in mood regulation were determined by computing Pearson's correlation coefficients (correlations were considered significant at Pearson's r≥0.8, which corresponds to a one-tailed significance level of *P*≤0.005 (Flagel et al., 2011; Moreno-Fernández et al., 2017; Wheeler et al., 2013). Each complete set of interregional correlations was plotted in colour-coded correlation matrices using MATLAB software (MathWorks, Natick, MA, USA). Network graphs were generated by considering only the strongest correlations (Pearson's r≥0.8, conservative thresholds). Finally, the degree of connectivity in naïve WT and maLPA_1_-null mice and in each experimental group was calculated by summing the absolute interregional correlations extracted from each matrix, as a simplified adaptation of the rich-club coefficient (Colizza et al., 2006).

A PCA was subsequently performed on c-Fos activation in the brain to characterise the functional network by reducing the complete, multidimensional set of regional activity data to a smaller set of dimensions underlying the animals' behaviour (Budaev, 2010; Castilla-Ortega et al., 2011, 2014). We used the Kaiser-Meyer-Olkin (KMO) test to test whether the PCA met the statistical adequacy criteria (Balaban et al., 2014). Then, because the component or factor scores represent the relative contribution or weight of each loading pattern for each case, Student's *t*-test was used to examine differences in each loading pattern between WT and maLPA_1_-null mice and between veh- and antagonist-treated animals. Finally, the degree of connectivity in each network, based on the factors resulting from PCA analysis, was calculated for every treatment, and highly connected regions were identified (Wheeler et al., 2013).

## Supplementary Material

Supplementary information

First Person interview
