# Supplementary material for: Effects of genetic deletion versus pharmacological blockade of the LPA1 receptor on depression-like behaviour and related brain functional activity
Source: Dis Model Mech. 2018 Sep 10;11(9):dmm035519. doi: 10.1242/dmm.035519 (PMC6177006; doi:10.1242/dmm.035519)
Supplement: Supplementary information [file dmm-11-035519-s1.pdf]

FIRST PERSON

First person – Román Darío Moreno-Fernández

First Person is a series of interviews with the first authors of a selection of papers published in Disease Models & Mechanisms, helping early-career researchers promote themselves alongside their papers. Román Darío Moreno-Fernández is first author on 'Effects of genetic deletion versus pharmacological blockade of the LPA_1 receptor on depression-like behaviour and related brain functional activity', published in DMM. Román is a PhD student in the lab of Carmen Pedraza at Universidad de Málaga, Málaga, Spain, investigating stress, depression and the role of the LPA_1 receptor.

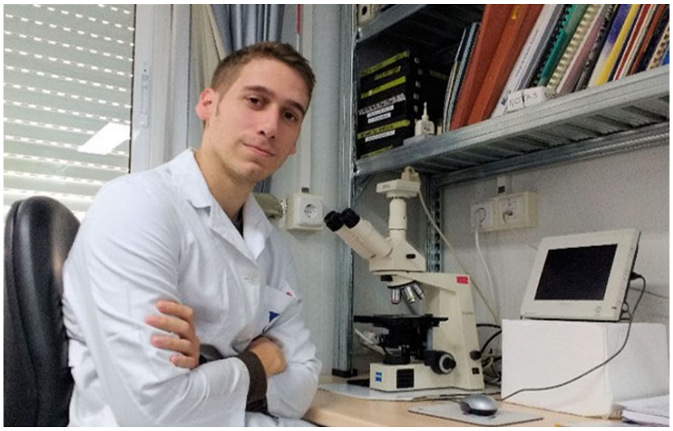

Román Darío Moreno-Fernández

How would you explain the main findings of your paper to non-scientific family and friends?

In order to understand the brain of a person who suffers from depression, there are several approaches a research group can take, particularly in rodent studies. In our group, thanks to the development of a colony of mice without a key gene for the brain response to stress, the gene for the LPA_1 receptor, we have identified that this absence is in fact related to several features described in human depression, such as loss of interest in pleasurable activities (e.g. sweet taste or sexual incentives), or specific patterns of brain activation and connectivity when exposed to stressful events. However, not having one gene expressing an important protein engaged in normal brain development throughout the lifespan can complicate the interpretation of these results: what is behind the depression-like symptoms and the abnormal brain response? The specific lack of the LPA_1 receptor in the brain or the compensatory mechanisms and brain adaptations derived from this genetic absence? To address this issue, we included a group of mice that had a normal development but received a drug that centrally blocks the LPA_1 receptor (see figure). We discovered that, yes, the pharmacological approach also induced some of the symptoms displayed in the genetic approach, but there are also significant differences between the experimental groups in the way the brain responded to stress, which could account for the divergent behavioural strategies when facing a stressful situation.

What are the potential implications of these results for your field of research?

Our findings not only enrich the current knowledge of the relationship between the LPA_1 receptor and depression, but also highlight the importance of using different and complementary approaches to the study of the underlying neurobiology of depression in mouse models. A decrease in the expression of the LPA_1 receptor gene has been previously reported in the temporal lobe of the brain of depressed patients, and the expression of this gene was used as a biomarker of high mood after cognitive behavioural therapy for depression. In this sense, our study sheds light on the brain activity changes induced by the depletion or blockade of the LPA_1 receptor and identifies this factor as a potential brain target responsible for some of the depressive symptomatology related to stress, a well-known environmental factor involved in the onset of depression. Additionally, this work provides further insight and considerations in the use of knockout models.

Román Darío Moreno-Fernández's contact details: Departamento de Psicobiologia y Metodologia en las CC, Instituto de Investigación Biomédica de Málaga (IBIMA), Universidad de Málaga, Málaga 29071, Spain.
E-mail: roman.moreno@uma.es

"Our findings not only enrich the current knowledge of the relationship between the LPA_1 receptor and depression, but also highlight the importance of using different and complementary approaches to the study of the underlying neurobiology of depression in mouse models."

What are the main advantages and drawbacks of the model system you have used as it relates to the disease you are investigating?

First, using two separate approaches (genetic vs pharmacological) and observing the effect on both brain and behaviour represent a step forward in the research of specific depressive phenotypes. In addition, we examined two of the most used behavioural tasks in mouse models of depression – the forced swim test (FST) and the tail suspension test (TST) – and took the FST as an acute stressor to examine brain response among the four experimental groups through quantification of *c-Fos*, an immediate early gene. However, through this type of quantification we can only see general brain activation and interregional connectivity, but have not examined the specific profile of neurotransmission between brain areas. For example, since we are dealing with the neurobiological basis of depressive behaviours, it could be particularly relevant to analyse whether the differences in the dorsal raphe nucleus or ventral tegmental area are related to dysfunction of the serotoninergic or dopaminergic neurotransmission systems, respectively.

What has surprised you the most while conducting your research?

For me, the most striking result was the behaviour exhibited by the antagonist-treated mice in FST and TST, which performed in an opposed matter in two apparently similar tasks. That moment is when I remember the sentence 'The TST is more than just a dry-land version of the FST', written in a subheading of a review by Cryan et al. (2005). One possible explanation could be the different neurobiological pathways in these two models described by

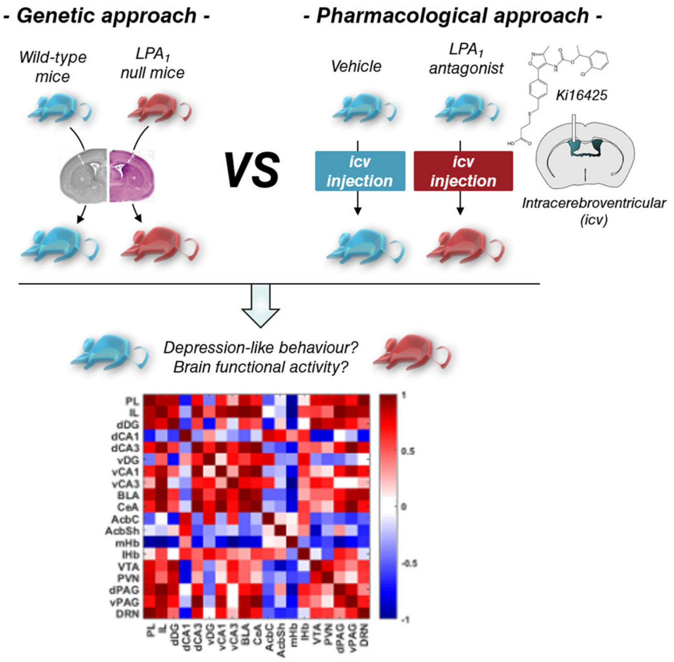

Representative summary of the rationale behind the study.

previous research. However, in the literature, both tasks are frequently used interchangeably.

What changes do you think could improve the professional lives of early-career scientists?

The welcome cocktail of underpaid temporary research positions with no weekends, together with ‘publish or perish’ pressure, unemployed periods and unreachable postdoc evaluation criteria makes early career scientists wonder whether this was the adult life they were dreaming of when they were children. Given the socioeconomic and public health benefits of research, the financial support of a more stable scientific trajectory by the government could reduce the waste of highly qualified and talented professionals and eventually make the welcome (alcohol-free) cocktail a bit more appealing to new generations.

What’s next for you?

Presenting my PhD thesis and applying for postdoctoral funding.

Reference

Moreno-Fernández, R. D., Nieto-Quero, A., Gómez-Salas, F. J., Chun, J., Estivill-Torrús, G. Rodríguez de Fonseca, F., Santín, L. J., Pérez-Martín, M. and Pedraza, C. (2018). Effects of genetic deletion versus pharmacological blockade of the LPA_1 receptor on depression-like behaviour and related brain functional activity. *Dis. Model. Mech.* **11**: dmm035519.
